# The eutectic mixture local anesthetics (EMLA) cream is more effective on venipuncture pain compared with lidocaine tape in the same patients

**DOI:** 10.1186/s40981-018-0210-1

**Published:** 2018-10-08

**Authors:** Tomomi Matsumoto, Tomohiro Chaki, Naoyuki Hirata, Michiaki Yamakage

**Affiliations:** 1 Department of Anesthesiology, Tomakomai City Hospital, 1-5-20, Shimizu-cho, Tomakomai, Hokkaido Japan; 2 Department of Anesthesiology, Sapporo Medical University School of Medicine, 291, South 1, West 16, Chuo-ku, Sapporo, Hokkaido Japan

**Keywords:** Anesthesia, Local, Catheterization, Peripheral, Pain management

## Abstract

**Introduction:**

Although venous cannulation is imperative during perioperative period, it inevitably causes venipuncture pain. Eutectic mixture local anesthetics (EMLA) has been used to reduce this pain, and various studies have been conducted to evaluate the efficacy of EMLA. But these studies did not elucidate the effect of EMLA exactly, because there were large individual differences in pain sensitivity. The aim of this study is to accurately evaluate the efficacy of EMLA cream for venipuncture pain relief compared with lidocaine tape in the same patients.

**Methods:**

Participants were randomly allocated into EL or LE group. Participants received EMLA cream at one side dorsum of hand and lidocaine tape at another dorsum of hand before entering operation room. Local anesthetics were strictly applied according to their manufacturers’ instruments, respectively. In the EL group, participants received venipuncture at EMLA cream site firstly. In LE group, participants, conversely, received venipuncture at lidocaine tape site firstly. Before anesthetic induction, local anesthetics were removed followed by venous cannulations. After cannulation, participants evaluated the pain by visual analog scale (VAS) and verbal rating scale (VRS).The primary outcome was VAS, and the secondary outcome was VRS.

**Results:**

Data from 24 patients were analyzed. The VAS of EMLA cream was significantly lower than that of lidocaine tape (4 [0–18] vs 17 [8–45], *p* = 0.001, 95% CI − 25 to − 6). The VRS of EMLA cream was also significantly lower than that of lidocaine tape (2 [1–2] vs 2 [2–3], *p* = 0.002, 95% CI − 0.8 to − 0.2). The local skin adverse events were observed in five patients at EMLA cream applied hands.

**Conclusions:**

We conducted a comparative study to elucidate the efficacy of EMLA cream for venipuncture-pain comparing with lidocaine tape in the same patients. Our results strongly suggest that EMLA cream is more effective for venipuncture pain relief than lidocaine tape.

**Trial registrations:**

UMIN Clinical Trials Registry, UMIN000023030. Registered 5 July 2016.

## Background

Venous cannulation is a mandatory procedure for hydration and drug intravenous administration during the perioperative period. However, this procedure must be accompanied by pain. Various methods have been devised to attenuate this pain, one of which is the application of a eutectic mixture of local anesthetics (EMLA) cream. Although few decades have passed since the start of clinical usage of EMLA cream and several clinical studies evaluating its efficacy have been published [1], in all of these studies, the participants were divided into two groups and each group received only one of the study interventions. However, since there are large individual differences in pain sensitivity, even with the same stimuli, this kind of clinical study design lacks accuracy in pain evaluation [2]. The aim of this study was to precisely elucidate the efficacy of EMLA cream for venipuncture pain relief compared with lidocaine tape, a traditional analgesic method, in the same patients.

## Methods

**Fig. 1:**
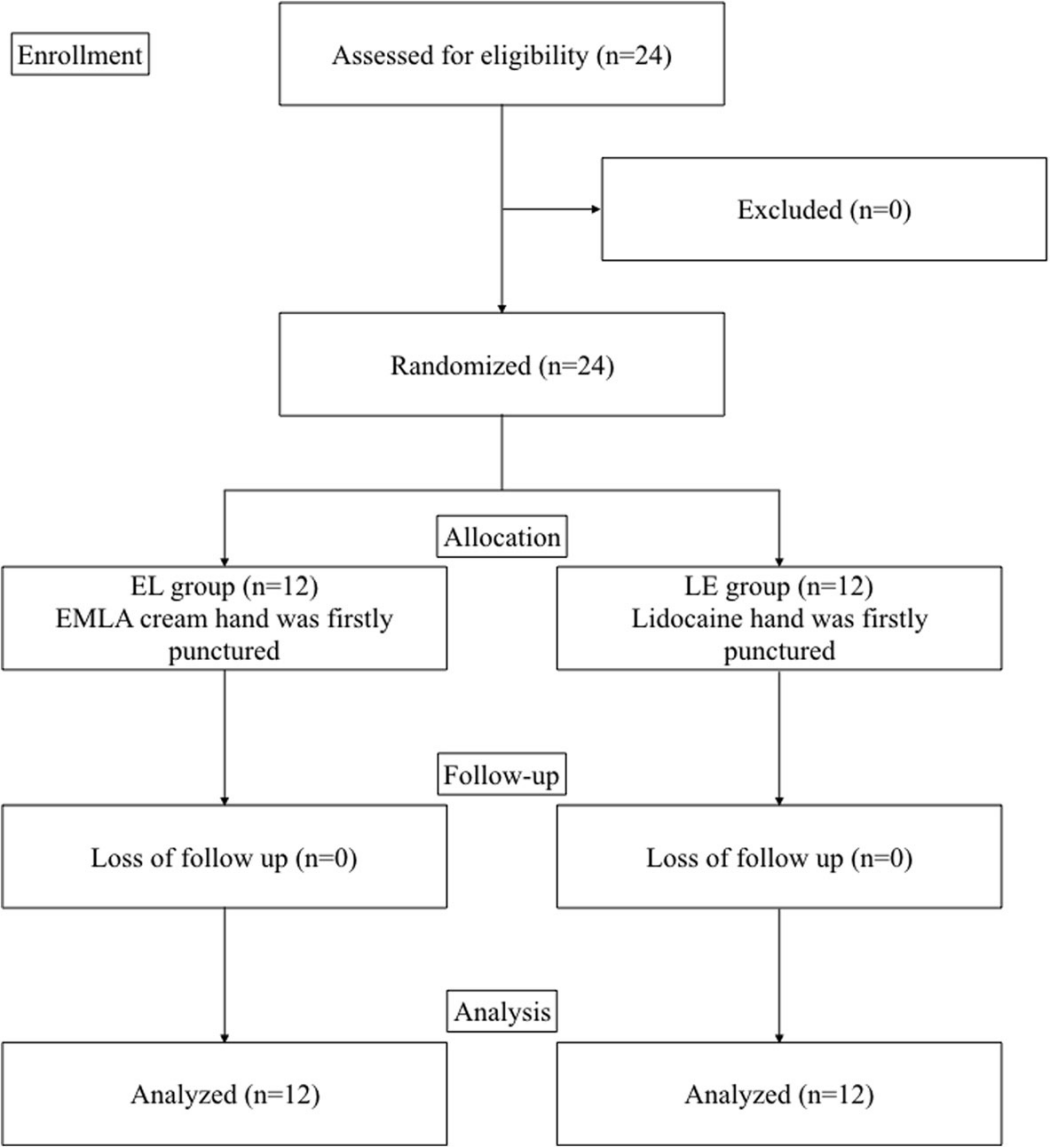
CONSORT flow diagram. EL group received venipuncture at EMLA cream site firstly and at lidocaine tape site secondarily. Conversely, LE group received venipuncture at lidocaine tape site firstly and at EMLA cream site secondarily. EMLA eutectic mixture of local anesthetics

This single-center, prospective, randomized, interventional study was conducted at Tomakomai City Hospital, Hokkaido, Japan, from July 2016 to March 2017. The study was approved by the institutional review board of Tomakomai City Hospital, Japan (approval code: 1605) and was registered in the UMIN-Clinical Trials Registry (UMIN trial ID: UMIN000023030). Written informed consents were received from all patients before participation in this study. Eligible participants were patients aged 16 years and above, American Society of Anesthesiologists physical status I–III, who required two venous access lines during elective surgery under general anesthesia. Exclusion criteria were patients who had local anesthetic allergy and skin abnormalities at the site of venipuncture. Patients with severe hepatic, renal, and cardiac diseases which might influence drug metabolism were also excluded. According to manufacturers’ instructions strictly, 1 g of 5% EMLA cream®, containing 25 mg of lidocaine and 25 mg of prilocaine per gram (Sato Seiyaku, Tokyo, Japan), was applied on the dorsum of one hand and covered by Tegaderm™ transparent film dressing (3 M Medical, Maplewood, MN) 60 min before entering the operation room, while a lidocaine tape (YouPatch tape® 18 mg, Yutoku Yakuhin, Saga, Japan) was applied on the dorsum of the other hand 30 min before operating room entrance for all participants. All participants were randomly allocated, according to computer randomization, to EL group: received venipuncture at EMLA cream site firstly and lidocaine tape site secondarily, or LE group: received venipuncture at lidocaine tape site firstly and EMLA cream site secondarily (Fig. 1). The timings and doses of local anesthetic applications were decided according to their respective manufacturers’ instructions in a rigorous manner. Just before anesthetic induction, the tape and cream were removed and two 20 gauge Surflo® ETFE intravenous catheters (Terumo Corp, Tokyo, Japan) were inserted at the locally anesthetized areas one by one. In all the patients, the order of venous cannulations followed the allocated group manner. After two venous cannulations, participants rated pain during the intravenous cannula insertion on a visual analog scale (VAS 0–100 mm; 0 mm, no pain; 100 mm, intolerable pain) and verbal rating scale (VRS 1–4; 1, no pain; 4, intolerable pain). The condition of the skin at the site of local anesthetic application was also observed to evaluate any side effects of the intervention (e.g., skin flare, color change).

### Outcomes

The primary outcome was the VAS score for venipuncture pain. The secondary outcomes were VRS score for venipuncture pain and the frequency of adverse effects, including cutaneous symptoms and local anesthetic toxicity. Moreover, subgroup analysis and comparison of first vs second venipuncture pain intensities were also performed as secondary outcomes.

### Statistical analysis

Both VAS and VRS scores, subgroup analysis, and the comparison of first and second puncture pain intensities in all participants were analyzed by the Wilcoxon signed-rank test, while the comparisons of first and second puncture pain intensities at EMLA and lidocaine sites were analyzed by the Mann-Whitney *U* test. The frequency of adverse effects was analyzed by the Fisher’s exact test. All statistical analyses were performed using GraphPad Prism 7.0 (GraphPad Software, La Jolla, CA). Data were presented as median [interquartile range]. *A p* value less than 0.05 was determined statistically significant. The sample size was calculated by G^*^power 3.1 (Heinrich-Heine-University, Düsseldorf, Germany). A VAS difference of 18.1 was considered as a clinically meaningful difference, based on the results of the study by Çelik et al. [3]. The sample size was estimated by the ability to detect a reduction in VAS scores of 18.1 with a standard deviation of 17.9 for EMLA cream and 10.6 for lidocaine tape with a two-sided 5% significance level and power of 0.8. The sample size calculation indicated that 24 patients were needed in this study.

## Results

**Fig. 2:**
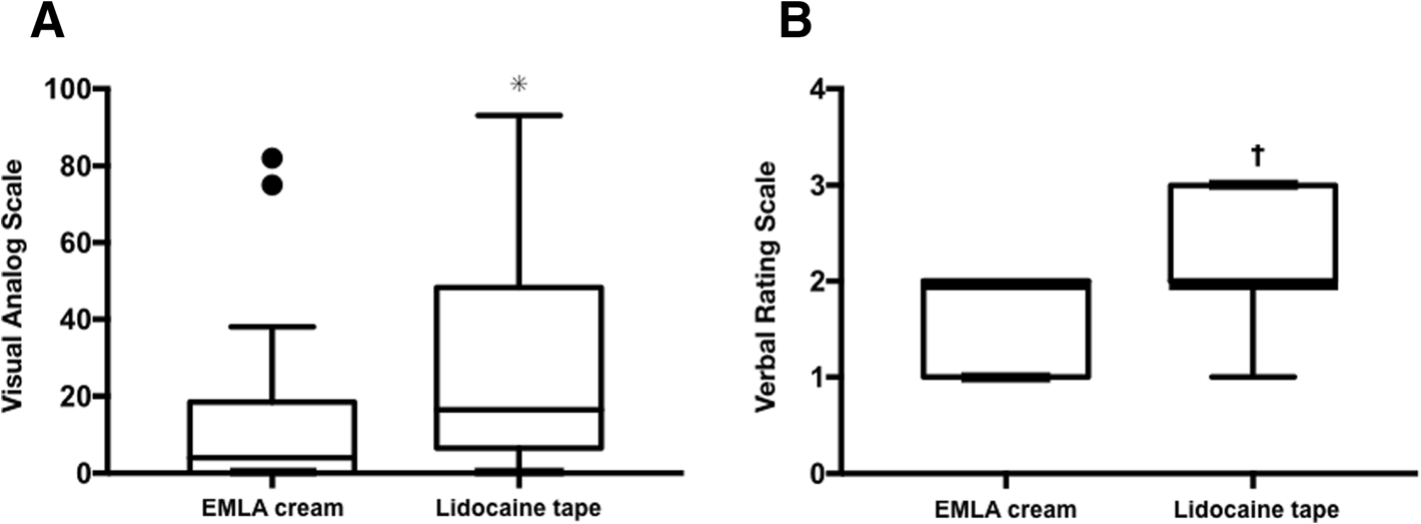
Comparison of pain intensities between EMLA cream and lidocaine tape. **a** Visual analog scale scores for pain during venous cannulation. *p = 0.001. **b** Verbal rating scale scores for pain during venous cannulation. †*p* = 0.002. Data are presented as median [interquartile range]. EMLA eutectic mixture of local anesthetics

A total of 24 patients were screened for inclusion in this trial, and all of the participants were included in this study. The 12 patients were allocated into EL group and other 12 patients were allocated into LE group. The characteristics of all participants are presented in Table 1. VAS scores for the EMLA cream hand were significantly lower than those for the lidocaine tape hand (4 [0–18] vs 17 [8–45], *p* = 0.001, 95% CI − 25 to − 6) (Fig. 2a). VRS scores for the EMLA cream hand were also significantly lower than those for the lidocaine tape hand (2 [1–2] vs 2 [2–3], *p* = 0.002, 95% CI − 0.8 to − 0.2) (Fig. 2b). Subgroup analysis revealed that the VAS and VRS in EL group and the VAS in LE group were statistically significantly lower in EMLA site than in lidocaine site, while there was no statistically significant difference in the VRS in LE group between EMLA and lidocaine (Table 2). There were no statistically significant differences in the comparison of first and second puncture pain intensities (Table 3). The frequency of local skin adverse event was significantly higher in EMLA cream (EMLA 5/24, 21% vs lidocaine 0/24, 0%, *p* = 0.022, relative risk 2.32, 95% CI 1.24 to 10.51). In detail, four of 24 hands that received the EMLA cream became paler following EMLA cream application, and one of 24 hands developed a skin flare with Tegaderm™ application, although these skin changes disappeared by the next day. No local adverse event was observed in lidocaine received hands. None of the patients experienced systemic adverse events in response to local anesthetic application, including symptoms of toxicity.

**Table 1:** Patient characteristics

Variable	
Number of patients	*n* = 24
Age (years)	70.0 [57.5–74.6]
Height (cm)	161.1 [155.6–167.0]
Weight (kg)	55.3 [49.9–61.5]
ASA PS (I/II/III)	2/14/8

Data are presented as median [interquartile range] and absolute number

**Table 2:** Subgroup analysis for pain intensity

	EMLA	Lidocaine	95% CI	*p* value
EL group	*n* = 12	*n* = 12		
VAS	2 [0–21]	25 [10–55]	− 30.6 to − 2.5	0.022
VRS	2 [1–2]	2 [2–3]	− 1.2 to − 0.3	0.016
LE group	*n* = 12	*n* = 12		
VAS	7 [0–19]	14 [4–42]	− 30.0 to − 0.5	0.022
VRS	2 [1–2]	2 [1–2]	− 0.7 to 0.1	0.250

Data are presented as median [interquartile range] and absolute number. *EMLA* eutectic mixture of local anesthetics, *VAS* visual analog scale, *VRS* verbal rating scale

**Table 3:** Comparison of first and second puncture pain intensities

	First puncture	Second puncture	95% CI	*p* value
First vs second puncture in total patients	*n* = 24	*n* = 24		
VAS	9 [0–39]	12 [2–36]	− 12.6 to 10.6	0.805
VRS	2 [1–2]	2 [1–2]	− 0.6 to 0.2	0.363
First vs second puncture at EMLA site	*n* = 12	*n* = 12		
VAS	2 [0–21]	7 [0–19]	− 9 to 10	> 0.999
VRS	1 [1–2]	1 [1–2]	− 1 to 0	> 0.999
First vs second puncture at lidocaine site	*n* = 12	*n* = 12		
VAS	14 [4–42]	25 [10–55]	− 14 to 35	0.401
VRS	2 [1–2]	2 [2–3]	0 to 1	0.140

Data are presented as median [interquartile range] and absolute number. *EMLA* eutectic mixture of local anesthetics, *VAS* visual analog scale, *VRS* verbal rating scale

## Discussion

We evaluated the efficacy of local anesthesia with EMLA cream and lidocaine tape for venipuncture before induction of general anesthesia in the same patients. The study results indicate that EMLA cream is more effective for pain relief during venipuncture than lidocaine tape. The novelty of this study lies in the fact that it compares two local anesthetic methods in the same person. Although some previous randomized-control studies on EMLA cream do exist, their participants were divided into two groups, and each group received only one local anesthetic method [1]. As is well known, there are significant individual differences in pain sensitivity [2]. Hence, it is ideal that the same participant evaluates the effect on venipuncture pain relief with both EMLA cream and lidocaine tape. In addition, in our study, the number of hands anesthetized with EMLA cream that was punctured first was the same to the number of hands anesthetized with the lidocaine tape that were punctured firstly, at 12 hands each. These features of our study design increase the level of accuracy and validity of our results that EMLA cream is more effective for pain relief during venous cannulation than lidocaine tape.

As confirmed by our study, EMLA cream provides more effective pain relief than lidocaine tape. A possible reason for this difference in efficacy is that EMLA cream is a eutectic mixture of local anesthetics that exhibits high skin permeability. However, considering a previous report that EMLA cream is superior to 1% lidocaine infiltration for arterial cannulation [4], the mechanism of EMLA cream efficacy is not limited to its high permeability. While lidocaine needs to be dissolved in some solvent for its application as an external preparation, EMLA cream does not require a solvent and provides a high concentration of local anesthetics. These two factors, the high permeability and concentrations of local anesthetics, are the mechanisms by which EMLA cream is more effective than lidocaine tape.

In this study, subgroup analysis and the comparison of first and second puncture-pain intensities were performed to clarify the influence of puncture order on pain severity evaluation. The second puncture pain intensity was higher than the first puncture’s pain, although this difference was not statistically significant. Moreover, the VRS in LE group was not statistically significant. These results suggest that the second puncture pain might become more severe than the first puncture pain. According to past reports, there is a positive relationship between anxiety and pain in clinical settings [5], and anxiolytic drugs can decrease pain caused by medical procedures [6]. In this study, there was some possibility that the participants felt some anxiety caused by first venipuncture pain, resulting in exacerbating the second puncture pain intensity. But, we assessed that this influence on the result of this study could be dismissed because the computer-based randomization was performed to equate the number of firstly punctured local anesthetic site.

In terms of the safety of local anesthetics, local anesthetic toxicity is the most important problem. In our study, serum concentrations of lidocaine and prilocaine were not measured. Oni et al. measured the serum concentration of lidocaine after application of 30 g of 2.5% EMLA cream on facial and neck skin [7]. The average peak serum concentration of lidocaine was 0.44 μg/mL at 90 min after application, despite the removal of the EMLA cream 60 min after its application. Although this concentration was lower than the threshold of lidocaine toxicity, there were significant individual differences. The peak individual concentration of lidocaine in their study was 0.78 μg/mL, which is about twofold that of the average value. There are three major factors responsible for the individual differences in absorption and serum concentrations of local anesthetics. The first is body weight. Previous case reports regarding local anesthetic toxicity in 3-year-old children who received EMLA cream have been published [8, 9]. The application of EMLA in small children, therefore, requires strict attention. The second factor is liver function impairment. Local anesthetics, including lidocaine, are metabolized in the liver (e.g., lidocaine is metabolized into monoethylglycinexylidide by the liver and eliminated by the kidney). Strict attention is also needed during application of EMLA to patients with liver function failure. The last factor is skin disorders. The barrier function of the skin is lost in areas of skin with dermopathy, resulting in excessive absorption of local anesthetics. Juhlin et al. evaluated the plasma concentrations of lidocaine and prilocaine after application of EMLA cream and compared these concentrations between normal and diseased skin patients [10]. In diseased skin patients, the absorption was faster than in normal skin patients, resulting in high plasma concentrations. The peak concentrations of local anesthetics in patients with diseased skin were tens of times higher than those in patients with normal skin. These indicate the need for close attention to be paid when EMLA is applied in patients who have any of the above three factors that can lead to an increase in serum concentrations of local anesthetics, resulting in systemic toxicity of local anesthetics.

Another side effect of topical anesthetics is alteration of skin color, such as pallor and flare. EMLA frequently produces blanching of the skin by causing vasoconstriction [11]. Although this side effect tends to disappear within a few days, it makes venipuncture and venous cannulation difficult [12]. The factors affecting difficult peripheral venous cannulation were investigated by Fields et al. and Piredda et al. [13, 14]. They reported an association between difficulty in peripheral venous cannulation and diabetes, intravenous drug abuse, sickle cell disease, veins with many valves, venous fragility, visibility and palpability, and a history of chemotherapy received via the peripheral cannula. Hence, during venous cannulation in patients with these factors, it might be better to avoid the use of EMLA to facilitate successful cannulation.

### Limitations

This study has two major limitations. The first is that we evaluated pain caused by venous cannulation using only subjective measurements. To evaluate pain objectively, heart rate and blood pressure should be measured before and after venipuncture. But, these parameters were also influenced by emotional factor easily, and the accuracy for evaluating venipuncture pain was very limited. The second limitation is that the serum concentrations of lidocaine and prilocaine were not measured to elucidate the safety of EMLA cream and lidocaine tape. Although none of the patients expressed symptoms of local anesthetic toxicity in this study, the time course of serum concentrations of lidocaine and prilocaine should be measured until 90 min after the application of EMLA to confirm the safety of this drug [7].

## Conclusions

We performed this prospective comparative study to accurately elucidate the efficacy of EMLA cream and lidocaine tape on pain relief during venipuncture in the same patients. These results definitely indicate that EMLA cream provides more effective relief of the pain resulting from venous cannulation. A more detailed study is necessary to confirm the safety of EMLA cream in various patient groups and under different clinical conditions.

## Abbreviations

EMLA: Eutectic mixture local anesthetics
VAS: Visual analog scale
VRS: Verbal rating scale
